# A Review of Recent Findings on Meal Sequence: An Attractive Dietary Approach to Prevention and Management of Type 2 Diabetes

**DOI:** 10.3390/nu12092502

**Published:** 2020-08-19

**Authors:** Sodai Kubota, Yanyan Liu, Katsumi Iizuka, Hitoshi Kuwata, Yutaka Seino, Daisuke Yabe

**Affiliations:** 1 Department of Diabetes and Endocrinology, Gifu University Graduate School of Medicine, Gifu 501-1194, Japan; sodai0@gifu-u.ac.jp (S.K.); yyliu@gifu-u.ac.jp (Y.L.); kiizuka@gifu-u.ac.jp (K.I.); 2 Yutaka Seino Distinguished Center for Diabetes Research, Kansai Electric Power Medical Research Institute, Kobe 650-0047, Japan; kuwata-kob@umin.ac.jp (H.K.); yutaka.seino@kepmri.org (Y.S.); 3 Center for Diabetes, Endocrinology and Metabolism, Kansai Electric Power Hospital, Osaka 553-0003, Japan; 4 Division of Molecular and Metabolic Medicine, Department of Physiology and Cell Biology, Kobe University Graduate School of Medicine, Kobe 650-0047, Japan

**Keywords:** dietary therapy, meal sequence, diabetes, obesity, incretin

## Abstract

While adjustment of total energy and nutritional balance is critically important, meal sequence, a relatively simple method of correcting postprandial hyperglycemia, is becoming established as a practical dietary approach for prevention and management of diabetes and obesity. Meal sequence, i.e., consumption of protein and/or fat before carbohydrate, promotes secretion of glucagon-like peptide-1 (GLP-1) from the gut and ameliorates secretions of insulin and glucagon and delays gastric emptying, thereby improving postprandial glucose excursion. GLP-1 is known to suppress appetite by acting on the hypothalamus via the afferent vagus nerve. Thus, enhancement of GLP-1 secretion by meal sequence is expected to reduce body weight. Importantly, consumption of a diet rich in saturated fatty acids such as meat dishes before carbohydrate increases secretions of not only GLP-1 but also glucose-dependent insulinotropic polypeptide (GIP), which promotes energy storage in adipose tissue and may lead to weight gain in the long term. Dietary fiber intake before carbohydrate intake significantly reduces postprandial glucose elevation and may have a weight loss effect, but this dietary strategy does not enhance the secretion of GLP-1. Thus, it is suggested that their combination may have additive effects on postprandial glucose excursion and body weight. Indeed, results of some clinical research supports the idea that ingesting dietary fiber together with meal sequence of protein and/or fat before carbohydrate benefits metabolic conditions of individuals with diabetes and obesity.

## 1. Introduction

Early metabolic changes in type 2 diabetes (T2D) typically show an increase in postprandial blood glucose [1]. Postprandial hyperglycemia is an independent risk factor for complications of T2D, including microvascular and macrovascular complications [2,3,4]. Furthermore, it has been shown that decreasing postprandial glucose elevation can reduce the incidence of T2D and that controlling postprandial blood glucose with dietary therapy may be effective in preventing diabetes [5,6]. Under these circumstances, there is much interest in meal sequence for the control of postprandial glucose elevation and body weight. Meal sequence, a relatively simple method of correcting postprandial hyperglycemia, is becoming established as a practical dietary treatment for diabetes and obesity. In this article, the mechanisms involved in the beneficial effects of meal sequence are discussed.

## 2. Secretion and Function of Glucagon-Like Peptide-1 (GLP-1)

GLP-1 is one of two incretins secreted from the gut in response to ingestion of the various nutrients (e.g., carbohydrate, protein, and fat), and stimulate insulin secretion from pancreatic β-cells glucose-dependently [7]. In addition, GLP-1 suppresses glucagon secretion from pancreatic α-cells and delays gastric emptying, thereby ameliorating postprandial glucose excursion [7]. GLP-1 has been attracting interest as an important therapeutic strategy for diabetes, and GLP-1 receptor agonists are now being used to manage glycemia in individuals with T2D globally. GLP-1 is also known to suppress appetite and reduce food intake; and GLP-1 receptor agonists reduce body weight substantially, making the drugs valuable for individuals with morbid obesity [7]. Studies in experimental animals revealed that effects of GLP-1 on secretions of insulin and glucagon, gastric emptying and appetite involve activation of the vagus nerve [8]. Studies using GLP-1 receptor- deficient mice revealed that GLP-1 exerts protective effects on heart, kidney, and nervous system, suggesting that GLP-1 may prevent diabetes-related complications [9,10,11]. Indeed, some GLP-1 receptor agonists have been shown to reduce incidence of cardiovascular death, non-fatal myocardial infarction, non-fatal cerebral infarction, and impaired renal function in high-risk T2D patients [12,13]. Taken together, it is conceivable that enhancement of GLP-1 secretion by nutrients should exert beneficial effects on prevention and progression of diabetes and obesity.

## 3. Preloading Protein and/or Fats before Carbohydrates

Various studies have been conducted to investigate the effects of amelioration of postprandial blood glucose levels by preloading protein and amino acids before carbohydrate intake. For example, intake of 55 g whey protein before potato soup enhances GLP-1 and insulin secretion, delays gastric emptying and ameliorates postprandial glucose elevation [14]. Intake of 40 g glutamine before mixed meal (37 g carbohydrate, 1.3 g fat, and 16 g protein) enhances GLP-1 and insulin secretion and ameliorates postprandial glucose elevation as well [15]. These effects have been observed in T2D and nondiabetic subjects [16]. It was reported that the glucose-lowering effect of protein preload was dose-dependent [17]. Several studies have been conducted to investigate the effects of preloading fats. Gentilcore D et al. reported that intake of 30 mL olive oil before mashed potato (61 g carbohydrate) enhances GLP-1, delays gastric emptying and ameliorates postprandial glucose elevation [18]. However, enhancement of insulin secretion was not found in the study. The effect of delay in gastric emptying appears more strongly when preloading olive oil than when preloading whey proteins. Thus, inhibition of gastric emptying may ameliorate glucose elevation more strongly than enhancement of insulin secretion by incretins.

**Figure 1:**
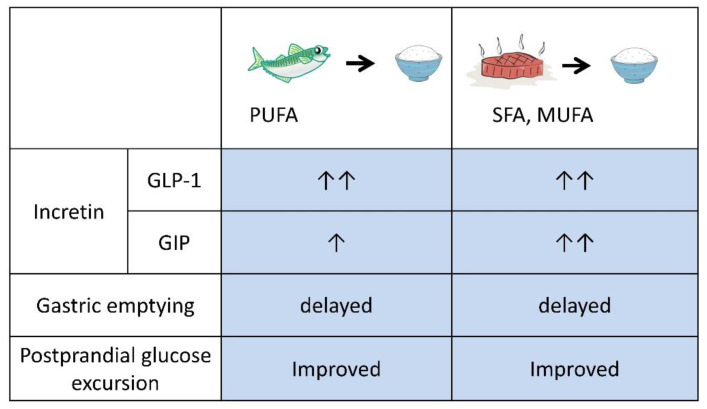
Summary of preloading fish and meat before carbohydrate. Preload of fish and meat ameliorated postprandial glucose excursion by enhancing GLP-1 secretion and delaying gastric emptying. Preload of meat, which is rich in saturated fat (SFA) and monounsaturated fat (MUFA), enhances glucose-dependent insulinotropic polypeptide (GIP) secretion more than fish, which is rich in polyunsaturated fat (PUFA). As GIP promotes fat accumulation, frequent preload of meat before carbohydrate might well increase body weight.

Preloading protein or fat before carbohydrate increased GLP-1 secretion and ameliorated postprandial hyperglycemia; however, intake of several tens of grams of whey protein, glutamine or olive oil at each meal, as performed in those protocols, is unrealistic. Thus, our group previously investigated preloading nutrients in daily meals. The effects of preloading fish (boiled mackerel: 15 g protein, 18 g fat, 0 g carbohydrate) and meat (grilled beef: 15 g protein, 18 g fat, 0 g carbohydrate) as a source of mixed protein and fat to be consumed before rice, as the source of carbohydrate, were evaluated in a crossover study comparing drug-naive T2D patients and healthy subjects (Figure 1) [19].

**Figure 2:**
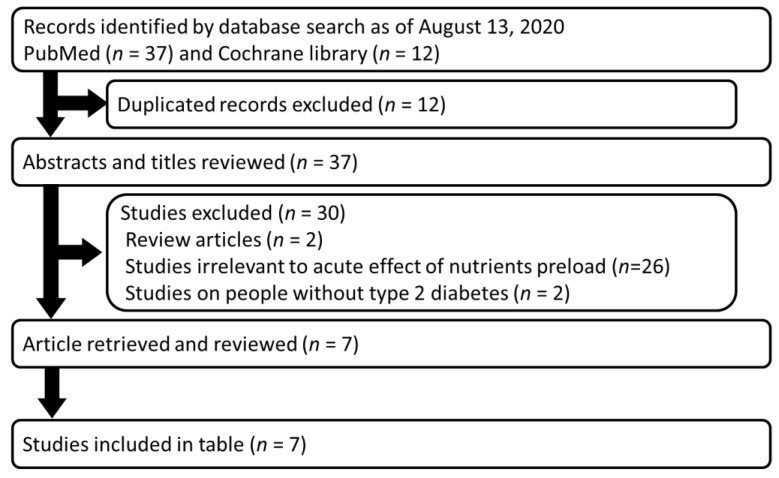
Flow diagram of the systematic review. Relevant articles were searched in PubMed and Cochrane library. The search terms were (Fat OR Protein OR fiber) AND (Preload OR “meal sequence” OR “postprandial glycemia” OR “postprandial glucose” OR “glucose excursion”) AND (“type 2 diabetes”) AND (“glucose-dependent insulinotropic polypeptide”) AND (“glucagon-like peptide-1”) NOT (Review[pt]). The resulting 37 articles were inspected for their relevancy, and 7 manuscripts are listed in Table 1.

When fish or meat dishes were consumed before rice, the postprandial glucose elevation was significantly reduced, and the secretion of GLP-1 was increased and gastric emptying time was prolonged. These findings are consistent with currently available data on preloading oil, protein, and their mixture with or without dietary fiber (Figure 2 and Table 1).

Interestingly, in a comparison between meat and fish dishes, a significant difference in secretion of another incretin, glucose-dependent insulinotropic polypeptide (GIP) was observed, although the energy content, nutrient ratio, and amino acid compositions were similar (Figure 1). Fish dishes contain more polyunsaturated fatty acids, eicosapentaenoic acid, and docosahexaenoic acid, while meat dishes contain more saturated fatty acids. The difference in fatty acids may underlie the difference in GIP secretion. It is known that saturated and monounsaturated fats can enhance GIP secretion in humans [23,24]. When a meal rich in saturated fatty acids is consumed, large amounts of GIP are secreted, which promotes energy storage in the adipose tissue [9]. Therefore, preloading meats, which are rich in saturated fatty acids, even if postprandial hyperglycemia is suppressed in the short term, may lead to weight gain in the long term.

## 4. Preloading Dietary Fiber before Carbohydrates

The effects of preloading dietary fiber before carbohydrate intake has also been discussed intensively. Dietary fiber is a generic term for components that are not digested and absorbed in the body. It has been shown that fiber has an effect on lifestyle-related diseases such as diabetes and obesity, through inhibition of carbohydrate and lipid absorption in the short term and through effects on gut microbiota in the long term [25]. It is believed that dietary fiber swells in the stomach, increasing the viscosity of the food mass and delaying the time of gastric emptying [26]; however, in our study, no obvious delay in gastric emptying time or increase in GLP-1 secretion when vegetables were ingested before rice was observed (S.K., H.K., and D.Y. unpublished observation). Sun et al. reports a similar effect under normal glucose tolerance, in which GLP-1 secretion shows no significant difference between eating vegetables before “meat and rice” and eating “vegetable, meat, and rice” together; however, glucose excursion was significantly ameliorated by eating vegetables before “meat and rice”. They found that ingesting vegetables first and meat afterward before rice was most ameliorative of postprandial glycemic excursion [27]. These results suggest that preloading protein or fat and dietary fiber before carbohydrate may have different mechanisms of effect on postprandial hyperglycemia and weight loss, and that the combination is expected to have additive effects. In addition, Jae Hyun Bae et al. reported the postprandial glucose-lowering effect of a premeal protein-enriched, dietary fiber-fortified bar containing a moderate amount of protein in individuals with T2D or normal glucose tolerance. The bar contained 0.4 g of carbohydrate, 9.3 g of whey protein, 1.4 g of soy protein, 0.3 g of fat, and 12.7 g of dietary fiber. The study showed a significantly decreased glucose elevation with a small amount of mixed protein and fiber [16].

## 5. Long-Term Effects of Preload-Based Dietary Strategies

**Figure 3:**
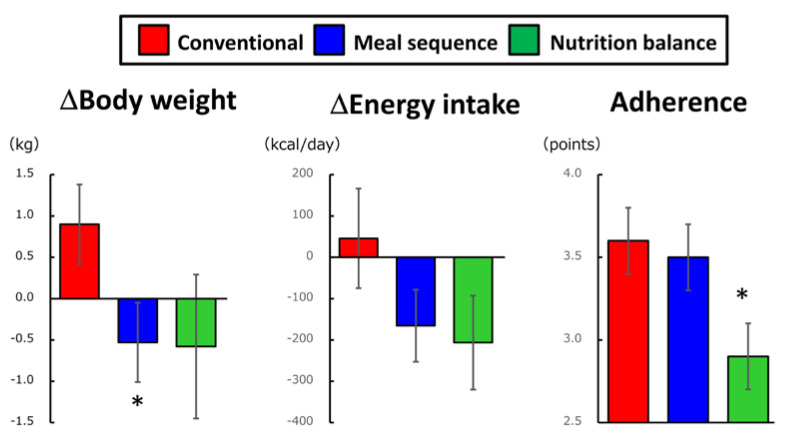
Dietary intervention focusing on meal sequence suppressed energy intake and reduced body weight in individuals with prediabetes. Effects of dietary instruction focusing on meal sequence (Meal sequence, *n* = 18) were compared to conventional dietary instruction (Conventional, *n* = 11) and dietary instruction focusing on nutritional balance (Nutrition balance, *n* = 13) using SMART Washoku^®^ (Kao Corporation, Tokyo, Japan), which can help individuals consume a more nutritionally balanced diet, in an exploratory, cluster-randomized, prospective, open-label, clinical trial [30]. Each participant reported adherence to their goals for diet and healthy exercise every month using a scale of 1–5, where 1 indicated “seldom adhered” and 5 indicated “fully adhered”. The group receiving dietary instruction focusing on meal sequence exhibited similar adherence and greater reduction in body weight than the group receiving conventional health guidance, while the group receiving dietary instructions focusing on nutritional balance failed to show significant body weight reduction, partly due to poor adherence. Mean±SEM; *, *p* < 0.05 versus conventional and *p* < 0.05 versus meal sequence.

A few reports have examined the long-term effects of preload-based dietary strategies. Toriko et al. studied 17 patients with type 2 diabetes divided into two groups: those who consumed protein and fat before carbohydrate and those who did not sequence meal components for 8 weeks. A significant decrease in HbA1c in the group that consumed protein and fat before carbohydrate was noted [28]. Imai et al. reported that Japanese T2D patients who were instructed to consume fiber-rich vegetables before carbohydrate showed a significant improvement in HbA1c and a trend toward decreased BMI [29]. Recently, the effects of dietary instructions focusing on meal sequence versus nutritional balance in individuals with prediabetes in the Japanese national health check-up and guidance program were recently evaluated. In this study, effects of health guidance with dietary instructions focusing on meal sequence were compared to conventional health guidance and health guidance with dietary instructions focusing on nutritional balance (Figure 3). It was found that health guidance with dietary instructions focusing on meal sequence reduced body weight compared to that with dietary instructions focusing on nutritional balance [30].

## 6. Conclusive Remarks and Future Perspectives

Many studies have found that preloading nutrients such as protein, fat, and fiber before carbohydrate can ameliorate postprandial glucose elevation. Preloading the various non-carbohydrate nutrients before carbohydrate intake engages distinct mechanisms but has a consistent effect on amelioration of elevated postprandial glucose. Interventions on the order of eating may be more readily followed than interventions on the nutritional balance of meals. Indeed, it has been reported that adherence to a meal sequence program is better than that to a nutritional balance program [30]. Interventions focusing on meal sequence facilitate secretion of GLP-1, which has the property of inhibiting appetite, suggesting that long-term interventions on meal sequence may lead to prevention or improvement of obesity. However, precise mechanisms underlying effects of meal sequence remains largely unknown. For example, it is unknown why consumption of protein and/fat before carbohydrate, but not after carbohydrate, enhances GLP-1 secretion. It is unknown whether other factors such as cholecystokinin and/or glucagon as well as the vagus nerve system that also regulates gastric emptying contribute to effects of gastric emptying. Experiments investigating mechanisms underlying meal sequence are underway. Meanwhile, currently available reports strongly support that meal sequence dietary therapy is beneficial in controlling postprandial glucose excursion and bodyweight to better prevent and manage diabetes and obesity.

## Figures and Tables

**Table 1:** Preloading fat, protein, and their mixture with and without dietary fibers before carbohydrate in individuals with type 2 diabetes.

Preload			Details of Main Meal	*n*	Outcomes							Ref
Type	Details	Timing			Glucose	Insulin	Glucagon	GLP-1	GIP	CCK	GE	
F	30 ml olive oil	30 min before C	65 g mashed potato/20 g glucose (C 61 g)	6	Peak delayed	Peak delayed	ND	Enhanced	NC	ND	Delayed	[18]
P	55 g whey protein	30 min before C	65 g mashed potato/20 g glucose (C 59.1 g; P 5.2 g; F 4.3 g)	8	Suppressed	Enhanced	ND	Enhanced	Enhanced	Enhanced	Delayed	[14]
P	25 g whey protein	30 min before C	65 g mashed potato/20 g glucose/1 egg yolk	22	Suppressed	Enhanced	Enhanced	Enhanced	Enhanced	ND	Delayed	[20]
Mixed	50 g cheese/one small-size boiled egg (C 2 g; P 23 g; F 17 g)	30 min before C	75 g glucose (C 75 g)	10	Suppressed	NC	Enhanced	Enhanced	Enhanced	ND	ND	[21]
Mixed	100 g steamed mackerel (C 0g; P 15.1 g; F 17.7 g)	15 min before C	150 g rice (C 53.4 g; P 3.5 g; F 0.6g)	12	Suppressed	Enhanced	Enhanced	Enhanced	Enhanced	ND	Delayed	[19]
Mixed	79 g grilled beef (C 0.2 g; P 16.4 g; F 17.1 g)	15 min before C	150 g rice (C 53.4 g; P 3.5 g; F 0.6g)	12	Suppressed	Enhanced	Enhanced	Enhanced	Enhanced*	ND	Delayed	[19]
Mixed	Protein enriched, dietary fiber fortified bar (C 0.4 g; P 10.7 g; F 0.3 g; Fiber 12.7 g)	30 min before C	286 g Bagle/70 g Cream cheese/95g Orange juice (C 79.5 g; P 15.5 g; F 50.5g)	15	Suppressed	Suppressed	ND	Enhanced	NC	ND	ND	[16]
Mixed	50 g cheese/one small-size boiled egg (C 2 g; P 23 g; F 17 g)	30 min before C	75 g glucose (C 75 g)	9	Suppressed	NC	Enhanced	Enhanced	Enhanced	ND	ND	[22]

Published results on preloading fat, protein, and their mixture with and without dietary fibers before carbohydrate in individuals with type 2 diabetes are summarized. C, carbohydrate; CCK, cholecystokinin; F, fat; GE, gastric emptying; GLP-1, glucagon-like polypeptide-1; GIP, glucose-dependent insulinotropic polypeptide; P, protein; NC, no change; ND, not determined. *, enhancement of GIP secretion was greater with preloading grilled meat compared to preloading steamed mackerel.
